# Lignite coal burning seam in the remote Altai Mountains harbors a hydrogen-driven thermophilic microbial community

**DOI:** 10.1038/s41598-018-25146-9

**Published:** 2018-04-30

**Authors:** Vitaly V. Kadnikov, Andrey V. Mardanov, Denis A. Ivasenko, Dmitry V. Antsiferov, Alexey V. Beletsky, Olga V. Karnachuk, Nikolay V. Ravin

**Affiliations:** 1 Institute of Bioengineering, Research Center of Biotechnology of the Russian Academy of Sciences, 119071 Moscow, Russia; 2 Laboratory of Biochemistry and Molecular Biology, Tomsk State University, 634050 Tomsk, Russia

## Abstract

Thermal ecosystems associated with underground coal combustion sites are rare and less studied than geothermal features. Here we analysed microbial communities of near-surface ground layer and bituminous substance in an open quarry heated by subsurface coal fire by metagenomic DNA sequencing. Taxonomic classification revealed dominance of only a few groups of *Firmicutes*. Near-complete genomes of three most abundant species, ‘*Candidatus* Carbobacillus altaicus’ AL32*, Brockia lithotrophica* AL31, and *Hydrogenibacillus schlegelii* AL33, were assembled. According to the genomic data, *Ca*. Carbobacillus altaicus AL32 is an aerobic heterotroph, while *B. lithotrophica* AL31 is a chemolithotrophic anaerobe assimilating CO_2_ via the Calvin cycle. *H. schlegelii* AL33 is an aerobe capable of both growth on organic compounds and carrying out CO_2_ fixation via the Calvin cycle. Phylogenetic analysis of the large subunit of RuBisCO of *B. lithotrophica* AL31 and *H. schlegelii* AL33 showed that it belongs to the type 1-E. All three *Firmicutes* species can gain energy from aerobic or anaerobic oxidation of molecular hydrogen, produced as a result of underground coal combustion along with other coal gases. We propose that thermophilic *Firmicutes*, whose spores can spread from their original geothermal habitats over long distances, are the first colonizers of this recently formed thermal ecosystem.

## Introduction

Studies of thermophilic microorganisms that survive and develop at temperatures that are extreme for ordinary life have broadened our understanding of the diversity of microorganisms and their evolution, the mechanisms of adaptation to environmental conditions. Most studies of thermophiles have been concentrated on thermal ecosystems associated with volcanic activity, such as hot springs and deep-sea hydrothermal vents, or with biotechnologically-related anthropogenic objects (high temperature bioreactors etc). In addition to volcanic activity, the processes of natural combustion of fossil hydrocarbons and coal can lead to the formation of natural thermal ecological niches. Thus, the phenomena of underground combustion of coal seams are quite widespread in nature and are found in Australia, Germany, the USA (Pennsylvania), China, Russia, India and other countries^1^. Such fires can last for centuries, such as coal seam in Dudweiler (Saarland, Germany), which arose in 1668. An example of long-term natural underground burning of coal is Burning Mountain in Australia^2^, the duration of which is estimated at about 6000 years.

Typically, the combustion of coal is accompanied by the release of large quantities of gas, which, in addition to CO_2_, contains hydrogen and carbon monoxide, as well as hydrocarbons^1,3^. These gases can also contain hydrogen sulfide, sulfur oxides, other toxic compounds such as benzene, xylene, aliphatic and halogenated compounds^3,4^. The sulfur compounds and other elements found in coal seams can be carried to the surface with a gas stream, which leads to contamination of surrounding areas^5^. The temperature of the coal gas emerging on the surface depends on the temperature at which underground coal combustion and the depth of the seams occur, and is usually in the range from 50 to 800 °C^1^.

In areas where hot coal gases come to the surface, local extreme ecosystems can be formed that are characterized by high temperatures (>50 °C) and the presence of toxic substances^6^. In addition, high-energy substances such as hydrogen and CO contained in the coal gases can be used by microorganisms as substrates, which determines the possibility of the development of specific communities of thermophilic microorganisms. Depending on the time of emergence of underground combustion, such thermal environments can have different ages and represent a model for studying the processes of colonization by microorganisms of new thermal ecosystems. However, very little is known about the diversity, composition and ecology of microbial communities of such ecosystems. In a single published paper^4^, the composition of microbial communities of soils around coal-fire gas vents in Xinjiang, China was studied using T-RFLP analysis and clone libraries of 16S rRNA genes, and the dominant groups of microorganisms were identified.

This study focuses on microbial communities associated with brown coal burning site in the Chagan-Uzun area of the Russian Altai Mountains. The Chagan-Uzun Valley is recognized for its history of extensive paleolakes^7^, as one of the largest flood event on Earth^8^, and also known for its seismic activity with the latest major earthquake in 2003^9,10^. The first reports of brown coal-bearing sediments in the area date back to the 19-th century with its later attribution to the Oligocene-Early Miocene formation^11^. The coal-bearing level consists of seven lignite seams with the maximum thickness of 3.67 m. The author refers to this lignite as the brownish-black sapropelic high ash coals containing gypsum crystals, pyrite, and amber. We are unaware of any reports of microbiological studies in this area.

In this work the microbial communities were studied by pyrosequencing of 16S rRNA gene fragments and metagenomic analysis, which resulted in obtaining near-complete composite genomes of dominating Bacteria.

## Results

### Elemental and mineralogical characteristic of the sampling sites

**Figure 1:**
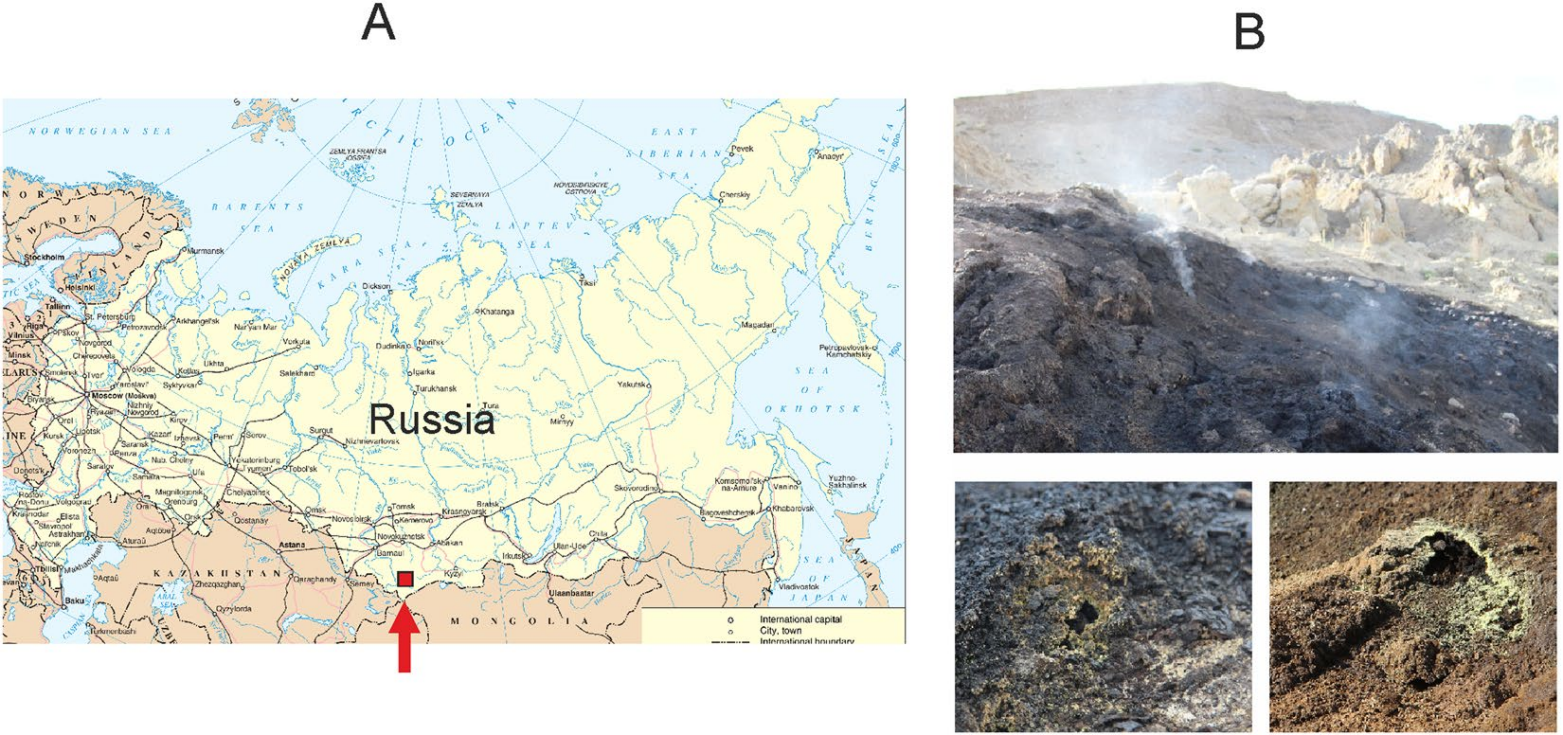
The site of underground combustion of brown coal studied in the work. (**A**) Location map. The red rectangle shows the location of the study area. The map was derived from a United Nations map (https://commons.wikimedia.org/wiki/File:Un-russia.png) which is in the public domain. (**B**) General view of the area where coal combustion occurs. The bottom part shows the fumarole-like structures. The images were taken by the authors.

Samples were collected from an abandoned lignite strip mining open pit near village Chagan-Uzun in Kosh-Agach district, Altai Mountains (Fig. 1A). The overburden was removed in late 1980s. The deposit has never been worked out due to high productive costs. Starting from the 1990s the underground fires in summer time were reported by locals. At the time of sampling signs of underground seam fire were visible as the intense heating of ground and rocks and raising clouds of steam with fumes from fumarole-like structures (Fig. 1B). The semi-liquid bitumen-like substance was discharged on one of the thermal spots.

The samples were collected from three different spots. AL3T was a ground sample in a heated area near the fumarole-like structure (Fig. 1B), while AL3B was a semi-solid sample collected at the bitumen-like discharge site. Bituminous substance in small quantities was also present in the AL3T sample. In addition the greyish-white crust on the fumarole surface was collected for elemental and mineralogical analysis only and designated AL3crust. The temperature at the sampling sites was between 50 and 70 °C.

**Figure 2:**
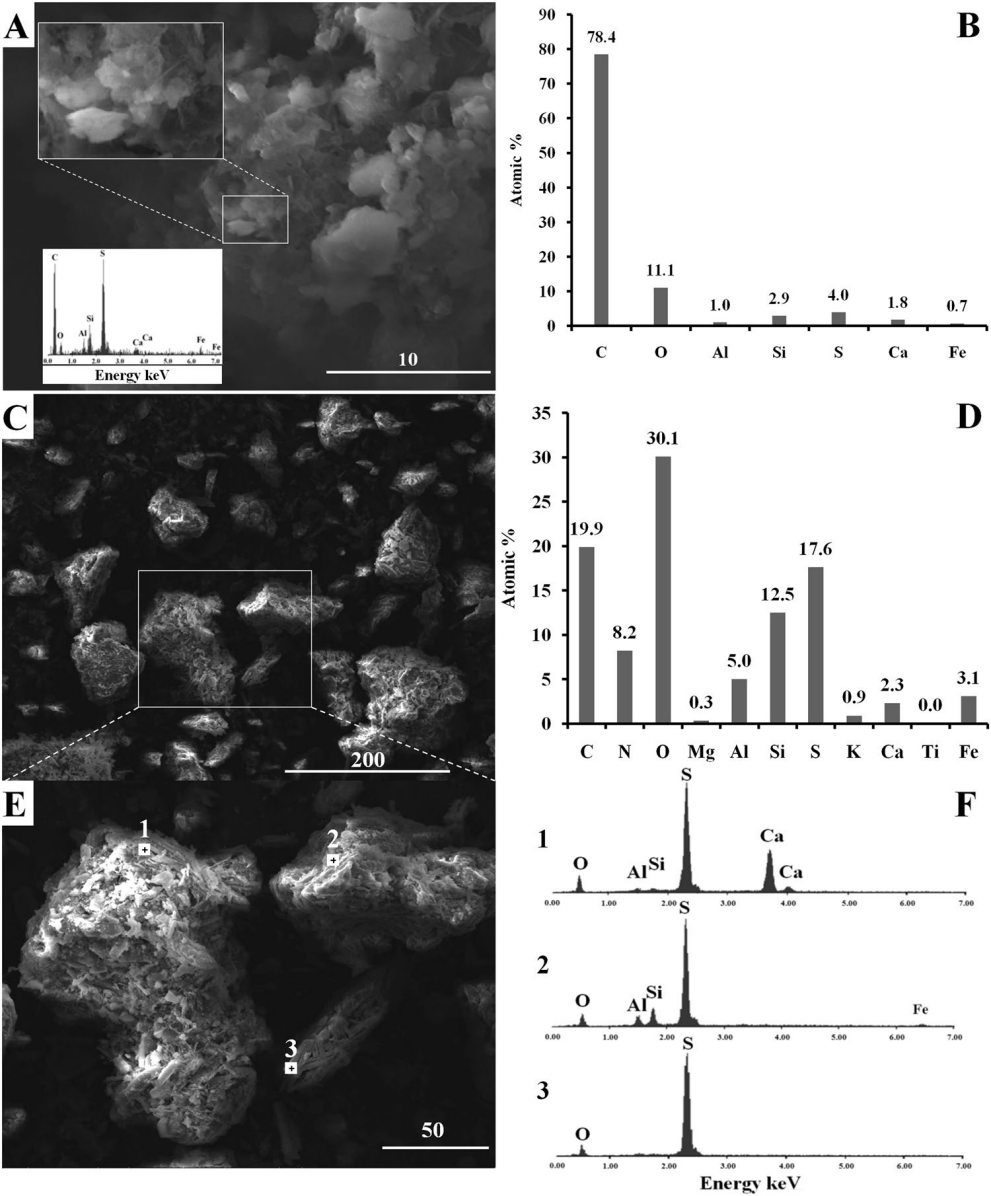
Characteristics of the chemical composition of the samples. Scanning electron micrographs of samples AL3T (**A**), and AL3crust (**C**), with the corresponding average atomic percentage of the prominent elements (**B** and **D**, respectively) calculated from EDS microprobes. An insert on panel A shows EDS spectrum of the S-enriched area, presumably amorphous sulphur. Panel E is an S-enriched area in AL3crust sample containing gypsum crystals (1), amorphous sulphur (2) and crystalline sulphur (3) in the locations 1, 2, and 3, respectively. Panel F shows the corresponding EDS spectra. Scale bars in the SEM images are in μm.

Electron microprobe analysis of sample AL3T showed high C and O content (Fig. 2A,B), which is to be attributed to the organic lithotypes of the lignite. Dry lignite contains about 60–57% carbon^12^ with the humic acids as one of the major components. Inorganic minerals detected in the brown coal and the combustion products included clays and silicates, as well as various sulfates (Table 1). Several of the detected sulfates, namely alunogen, mascagnite, boussingaultite, are known as secondary minerals associated with burning coal seams and fumaroles.

**Table 1:** Minerals identified by XRD in samples AL3T, AL3B, and AL3crust.

Mineral, phase	Formula
***Oxides and hydroxides***	
Hematite	Fe_2_O_3_
***Silicates/Clay***	
Quartz	SiO_2_
Kaolinite	Al_2_Si_2_O_5_(OH)_4_
Montmorillonite	Na_0.3_(Al, Mg)_2_Si_4_O_10_(OH)_2*_H_2_O
Muscovite	KAl_2_(AlSi_3_O_10_)(OH)_2_
Illite	(K_0.71_Ca_0.01_Na_0.01_)(Al_1.86_Mg_0.15_Fe_0.04_)(Si_3.27_Al_0.73_)O_10_(OH)_2_)
Clinochlore	(Mg, Fe, Al)_6_(Si,Al)_4_O_10_(OH)_8_
Wollastonite	CaSiO_3_
***Sulfates***	
Gypsum	Ca(SO_4_)(H_2_O)_2_
Epsomite	MgSO_4_(H_2_O)_7_
Hexahydrite	Mg(SO_4_)(H_2_O)_6_
Ferricopoapite	Fe_4.67_(SO_4_)_6_(OH)_2*_20H_2_O
Alunogen	Al_2_(SO_4_)_3*_17H_2_O
Mascagnite	(NH_4_)_2_SO_4_
Boussingaultite	(NH_4_)_2_(Mg(H_2_O)_6_)(SO_4_)_2_
Koktaite	(NH_4_)_2_Ca(SO_4_)_2*_(H_2_O)

The solid phases detected in the fumarole crust (sample AL3crust) contained numerous sulfates (Supplemental Fig. S1). There was less carbon as compared to the AL3T sample assuming that most of the organic matter was burnt out (Fig. 2C,D). The crust was rich in sulfur, which content was nearly 20%. Under scanning electron microscopy (SEM), amorphous sulfur, sulfur crystals and gypsum crystals were observed (Fig. 2E).

### Microbial community structures revealed by pyrosequencing of 16S rRNA gene fragments

**Figure 3:**
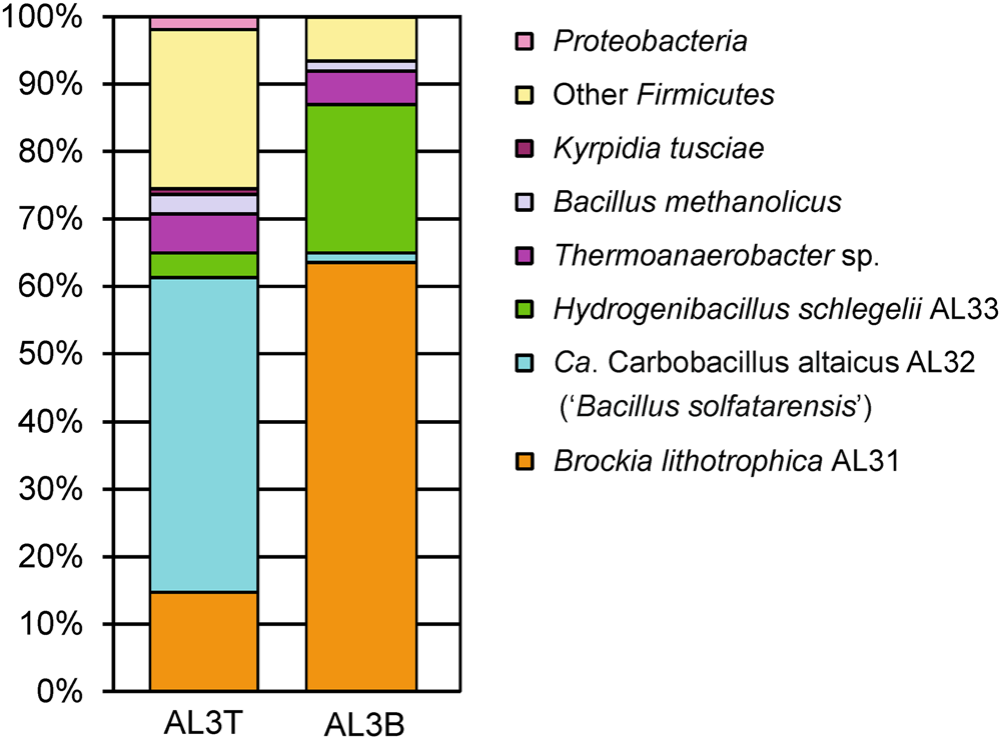
The relative abundance of taxonomic groups of microorganisms in the ground sample (AL3T) and in the bituminous substance (AL3B). Note that the name *Ca*. Carbobacillus altaicus AL32 was proposed for a newly identified lineage phylogenetically related to’*Bacillus solfatarensis*’.

A total of 6183 and 1834 16S rRNA gene sequences were obtained after applying quality filters for samples AL3T and AL3B, respectively. The results of the taxonomic classification of the obtained operational taxonomic units (OTUs) are shown in Fig. 3, and in more detail - in Supplemental Table S1. Archaea were absent in the microbial community of AL3T, which was dominated by *Firmicutes*, comprising more than 98% of the 16S rRNA gene sequences. About one half of the community bacteria were phylogenetically close to uncharacterized *Firmicutes* (GenBank accession number AY518549). The NCBI entry indicates that this bacterium called’*Bacillus solfatarensis*’ was isolated from La Solfatara, Naples, Italy. On the basis of the 16S rRNA gene sequence’*Bacillus solfatarensis*’ was considered not as a member of the genus *Bacillus*, but as a deeply branching lineage of *Bacilli* that warrants reclassification^13^.

The next most frequent community member (15% of 16S rRNA reads) was *Brockia lithotrophica*^14^. A single known strain of this species, Kam1851, was isolated from a terrestrial hot spring of the Uzon Caldera, Kamchatka Peninsula, Russia^14^. It is obligately anaerobic chemolithoautotrophic bacterium growing at temperatures between 46 and 78 °C with molecular hydrogen or formate as electron donors, and elemental sulfur, thiosulfate or polysulfide as electron acceptors^14^. The anaerobic part of the microbial community also included members of the genus *Thermoanaerobacter* (5.8%). Bacteria of this genus can grow organoheterotrophically by fermenting various sugars or using inorganic electron acceptors, in particular sulfur and thiosulfate. Some species, for example, *Thermoanaerobacter kivui*, can grow chemolithoautotrophically using molecular hydrogen and CO_2_ and form acetate as the product of CO_2_ reduction^15^. They perform autotrophic fixation of CO_2_ in the Wood-Ljungdahl pathway.

Two groups of aerobic thermophilic facultatively chemolitoautotrophic bacteria oxidizing molecular hydrogen were identified, - *Hydrogenibacillus schlegelii*^16,17^ and *Kyrpidia tusciae*^18,19^, accounting for 3.7% and 0.8% of 16S rRNA sequences, respectively. About 2.9% of 16S rRNA reads were assigned to *Bacillus methanolicus* (98% identity to 16S rRNA sequence of strain MGA3), aerobic thermophilic bacterium that can utilize methanol as its sole carbon and energy source^20,21^.

About 24% of microorganisms detected by the 16S rRNA genes sequences belonged to uncultured lineages of *Firmicutes*, phylogenetically remote from known species (less than 95% identity of 16S rRNA sequences). However, among the cultured species, the above-mentioned’*Bacillus solfatarensis*’, *Brockia lithotrophica*, *Hydrogenibacillus schlegelii*, as well as members of *Thermaerobacter*, were closest to these uncultured lineages with a similarity of 16S rRNA sequences in the range from 88 to 94%. Besides *Firmicutes*, only *Betaproteobacteria* of the order *Burkholderiales*, accounting for about 2% of all 16S reads were detected.

In the microbial community of the bitumen sample, AL3B, the same groups of bacteria were detected as in the ground sample, AL3T, but in other relative amounts ratios (Fig. 3, Table S1). In this community *B. lithotrophica* dominated, accounting for 64% of all 16S rRNA gene sequences obtained. The lineage related to ‘*B. solfatarensis*’, which constituted almost half of the microbial community in the ground sample, was found in a minor fraction in the bitumen (1.4%). Among the known species of *Firmicutes* in the sample AL3B we also detected *H. schlegelii* (22.0%), *Thermoanaerobacter* sp. (5.0%) and *B. methanolicus* (1.5%). The various uncultured lineages of *Firmicutes* accounted for about 6.5% of detected microorganisms, while *Proteobacteria* were present in small amounts (0.1%).

### Metagenome assembly and binning results

In order to assemble the composite genomes of the most numerous members of the microbial community, we sequenced the metagenome of the ground sample, AL3T. A total of 2.9 Gbp metagenomic sequences were assembled into contigs, which were distributed among 21 genome bins. More than 80% of metagenomics sequences corresponded to only three bins. Analysis of the presence of conservative single-copy marker genes with CheckM^22^ showed that two bins meets the recently proposed criteria for the high quality metagenome-assembled genomes (>90% completeness with <5% contamination)^23^ and one bin was slightly below this cutoff (Table 2). Full-length sequences of 16S rRNA genes were not found in these bins, however, the search for continuations of contigs representing fragments of 16S rRNA genes allowed to link unbinned 16S rRNA gene sequences to these three main bins.

**Table 2:** General characteristics of genomes obtained in this study.

Bin ID	AL31	AL32	AL33
Phylogenetic assignment	*Brockia lithotrophica*	*Ca*. Carbobacillus altaicus	*Hydrogenibacillus schlegelii*
Completeness (%)	90.64	89.74	93.97
Contamination (%)	0	6.15	0.79
Strain heterogeneity (%)	0	100	20
Contigs	12	236	110
Total length (bp)	1,689,796	3,083,259	2,735,157
Genome coverage	435	439	102
Protein-coding genes	1615	2942	2705
tRNA genes	50	51	43
16S rRNA gene copies per genome (estimate)	2	5	3
Aerobic respiratory chain	−	+	+
Calvin cycle	+	−	+
Uptake hydrogenase	+	+	+
CO dehydrogenase	−	−	+

Bin AL32, representing the genome of a bacterium which we designated ‘*Candidatus* Carbobacillus altaicus’ AL32 (lineage related to ‘*Bacillus solfatarensis*’), obtained with 439-fold coverage, accounted for the largest fraction of metagenome (46% of all reads). About 26% of the metagenomic sequences belonged to *B. lithotrophica*, which genome has 435-fold sequencing coverage (bin AL31). Comparison of the average coverage of the whole genome and the coverage of the 16S rRNA gene indicates that *Ca*. Carbobacillus altaicus AL32 has 5 copies of the 16S rRNA gene, while AL31 has only 2 copies, which explains three times the large share of *Ca*. Carbobacillus altaicus AL32 relative to *B. lithotrophica* observed in 16S rRNA profiling experiments. The third most abundant community member was *H. schlegelii*, which genome was obtained with 102-fold coverage (bin AL33).

**Figure 4:**
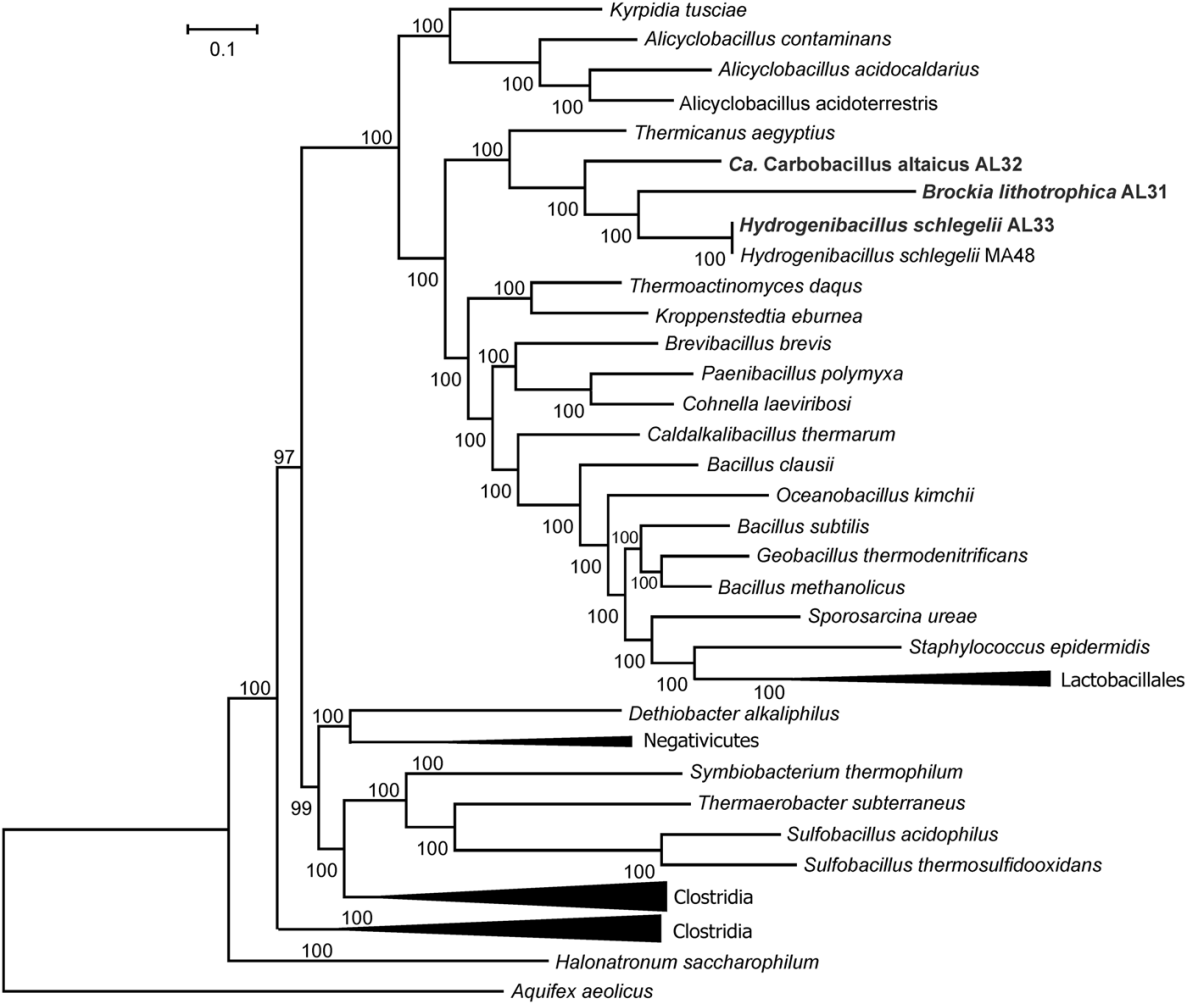
Position of the new genomes in the maximum likelihood concatenated protein phylogeny. The tree was inferred from the concatenation of 43 conserved marker genes and incorporates 59 genomes and 3 new genome bins. The support values for the internal nodes were estimated by approximate Bayes tests in PhyML. The scale bar represents the average number of substitutions per site.

To investigate the phylogenetic position of genomic bins, we constructed phylogenetic tree based on concatenated conservative marker genes. All three main bins (AL31, AL32 and AL33) together with *H. schlegelii* formed a distinct lineage close to the root of class *Bacilli* (Fig. 4).

To characterize the metabolic capabilities of the dominant microorganisms and their possible ecological roles in the community, we analyzed three composite genomes, preliminarily assigned to *B. lithotrophica*, *Ca*. Carbobacillus altaicus, and *H. schlegelii*. The genomes of the first two species were not previously sequenced.

### *B. lithotrophica* AL31: anaerobic hydrogenotrophic chemolithoautotroph

The composite genome of *B. lithotrophica* was obtained as 12 contigs longer than 2000 bp with N50 contig length of 279 kb. The size of the genome, estimated by the total length of contigs, is 1,689,796 nt and has a GC content of 64.3%. Based on the results of the genome annotation, 1615 protein-coding genes were identified, from which 1223 can be functionally assigned. The analysis of conservative single-copy marker genes with CheckM allowed estimating the completeness of the composite genome in 91% in the absence of contamination and heterogeneity (Table 2). A search of the full size 16S rRNA gene against GenBank showed that the closest relative of this bacterium is *B. lithotrophica* Kam1851 with 99.0% identity of the 16S rRNA sequences. Therefore, our *Brockia* represents a novel strain of the species *B. lithotrophica*, designated as AL31. Interestingly, other 16S rRNA sequences in GenBank (both among cultivated species and environmental clones) showed identity level of less than 90% indicating that *B. lithotrophica* is a phylogenetically isolated line, rarely found in the ecosystems studied to date.

**Figure 5:**
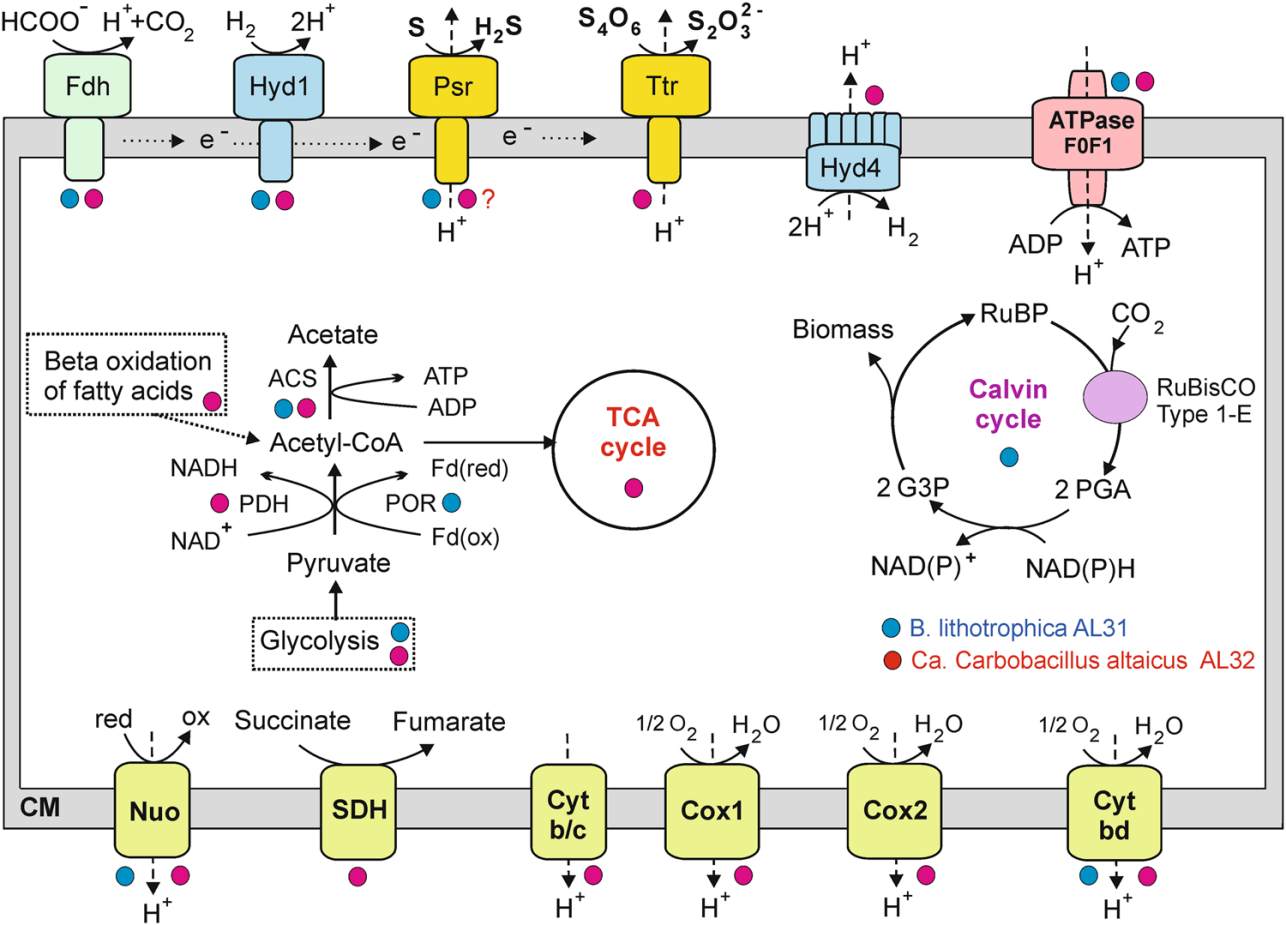
An overview of the metabolism of *B. lithotrophica* AL31 and *Ca*. Carbobacillus altaicus AL32 reconstructed from their genomes. Enzyme abbreviations: POR, pyruvate ferredoxin oxidoreductase; PDH, pyruvate dehydrogenase; ACS, acetyl-CoA synthetase; Fdh, formate dehydrogenase; Hyd1, membrane-bound group 1 [NiFe] uptake hydrogenase; Hyd4, membrane-bound group 4 f [NiFe] hydrogenase; Psh, polysulfide/thiosulfate reductase Ttr, tetrathionate reductase; ATPase F0F1, F_1_F_0_ – type ATP synthase; Nuo, membrane-linked complex comprising subunits NuoA, B, C, D, H, I, J, K, L, M and N of NADH-ubiquinone oxidoreductase; SDH, succinate dehydrogenase; Cyt b/c, cytochrome *b/c* complex, Cox, cytochrom *c* oxidase; Cyt bd, quinol oxidase *bd* complex. Other abbreviations: ox/red, oxidized and reduced forms; RuBP, ribulose-1,5-bisphosphate; PGA, 3-phosphoglycerate; G3P, glyceraldehydes-3-phosphate; CM, cytoplasmic membrane.

The microbiological characteristics of strain *B. lithotrophica* Kam1851 have shown that it is a spore-forming chemolithoautotroph, using hydrogen and format as electron donors, and elemental sulfur, thiosulfate or polysulfide as electron acceptors, which are reduced to H_2_S^14^. An overview of the central metabolic pathways of *B. lithotrophica* AL31, predicted by genome analysis, is shown in Fig. 5. Analysis of the genome revealed a set of genes of Embden-Meyerhof pathway for glycolysis and gluconeogenesis: glucokinase, glucose-6-phosphate isomerase, 6-phosphofructokinase, fructose bisphosphate aldolase, triose phosphate isomerase, glyceraldehyde-3 phosphate dehydrogenase, 3-phosphoglycerate kinase, phosphoglycerate mutase, enolase, and pyruvate kinase (gene numbers are provided in Supplemental Table S2). Taking into account the inability of strain Kam1851 to ferment sugars, the Embden–Meyerhof glycolytic pathway in *B. lithotrophica* probably operates in the direction of gluconeogenesis, as indicated by the presence of enzymes carrying out the reverse reactions: pyruvate, phosphate dikinase and fructose-1,6-bisphosphatase. Most genes of the pentose phosphate pathway are also present, including glucose-6-phosphate 3-dehydrogenase, 6-phosphogluconate dehydrogenase, ribulose phosphate 3-epimerase, ribose 5-phosphate isomerase, transketolase and transaldolase. We did not identify only the 6-phosphogluconolactonase gene of the oxidative stage of the pentose phosphate pathway.

Pyruvate could be reversibly decarboxylated to acetyl-CoA by pyruvate:ferredoxin oxidoreductase. The tricarboxylic acid (TCA) cycle in *B. lithotrophica* AL31 is incomplete, lacking succinyl-CoA synthetase, succinate dehydrogenase and malate dehydrogenase. This finding is consistent with the reported inability of strain Kam1851 to oxidize organic substrates completely.

**Figure 6:**
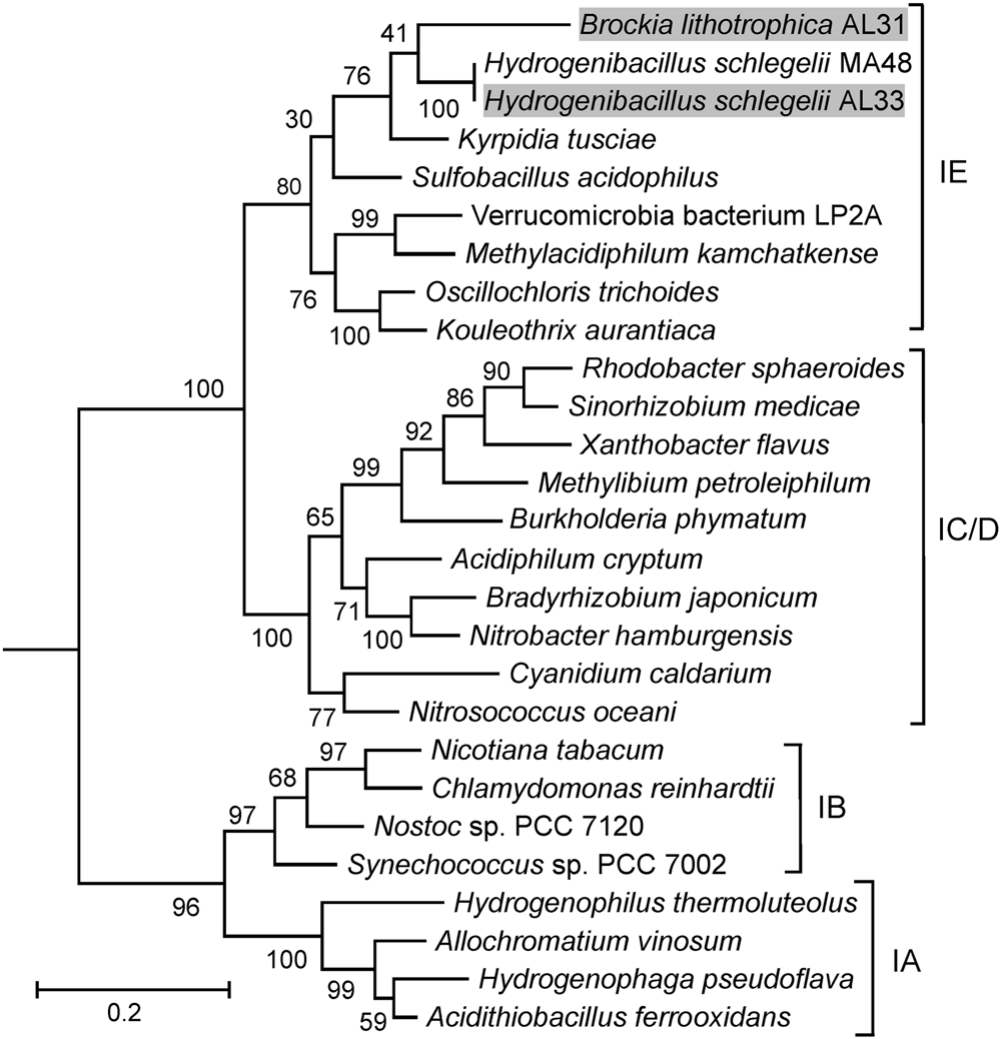
Maximum likelihood phylogenetic tree of deduced protein sequences of the large subunit of RuCisCO. Numbers at nodes represent bootstrap values (100 replications of the original dataset), only numbers above 50% are shown. The scale bar represents the average number of substitutions per site. The accession numbers for the sequences are given at the right side of species names.

Autotrophic carbon fixation could be performed through the Calvin cycle, including ribulose 1,5-bisphosphate carboxylase, phosphoribulokinase and enzymes of glucolytic and penthose phosphate pathways. The key enzymes of other known pathways of autotrophic carbon fixation, the reverse tricarboxylic acid cycle (citrate lyase) and the Wood-Ljungdahl pathway (carbon monoxide dehydrogenase/acetyl-CoA synthase), were not found. Phylogenetic analysis of the large subunit of RbcL showed that RuBisCO of *B. lithotrophica* AL31 belongs to the recently described type 1-E (Fig. 6)^24^. Among the *Firmicutes* this type of RuBisCo was found only in *K. tusciae, H. schlegelii, Sulfobacillus thermosulfidooxidans*, *Sulfobacillus thermotolerans* and *Sulfobacillus acidophilus*. All these species are thermophiles and are capable of autotrophic CO_2_ fixation, but they do not form a monophyletic group (Fig. 4). Therefore it can be assumed that at least some of these species have obtained RuBisCO genes as a result of lateral gene transfer from other *Firmicutes*, inhabiting the same thermal environment. The presence of *B. lithotrophica, K. tusciae, H. schlegelii* altogether in AL3T sample further supports this proposal.

An important feature of the metabolism of *B. lithotrophica* is its ability to use molecular hydrogen as an energy source. Analysis of the genome of strain AL31 revealed the presence of two of the [NiFe] -family hydrogenases of group 1, capable to oxidise H_2_ and transfer electrons to the quinone pool in the cytoplasmic membrane^25^. Each of the hydrogenases consists of a large, small and the third cytochrome *b* subunits linking them to the membrane. The presence of a Tat motif in the N termini of small subunits indicates that the hydrogenases are localized on the outer side of the cytoplasmic membrane. Along with molecular hydrogen, formate may be used as an electron donor by *B. lithotrophica* strain Kam1851, and the corresponding enzyme, formate dehydrogenase, was found in the genome of strain AL31.

Analysis of the genome of *B. lithotrophica* AL31 revealed a cluster of genes encoding the CISM oxidoreductase of the Psr/Psh family^26^, including the genes of molybdopterin-binding catalytic A subunit, the iron-sulfur electron transfer B subunit, and membrane subunit of the NrfD family. The catalytic A subunit containing the N-terminal Tat signal peptide is related to thiosulfate and polysulfide reductases. The presence of this enzyme, which can reduce thiosulfate and/or sulfur, can enable the use of these compounds as electron acceptors in chemolithoautotrophic growth of *B. lithotrophica* on hydrogen. Transmembrane proton gradient formed as a result of the activity of hydrogenases and molybdopterin oxidoreductase may be used for ATP synthesis by F_0_F_1_-type ATPase. Analysis of the genome did not reveal the pathway of dissimilatory sulfate reduction and other potential terminal oxidoreductases that could be used in aerobic or anaerobic respiration. The only encoded membrane-bound oxygen reductase, the cytochrome *bd*-type quinol oxidase, most likely provides oxygen detoxification.

### *Ca*. Carbobacillus altaicus AL32: facultatively autotrophic hydrogene-oxidising aerobe

Bin AL32 consisted of 236 contigs with a total length of 3,083,259 nt. Obtaining a large number of contigs in case of a high coverage of the genome probably results from the heterogeneity of this population, which is estimated by CheckM to be 100%. As a result of the genome annotation, 2942 protein-coding genes were identified from which 69% can be functionally assigned. The sequence of the 16S rRNA gene corresponding to this genome bin had the highest similarity (95.4% identity) to 16S rRNA of unvalidated species ‘*Bacillus solfatarensis*’, phylogenetically unrelated to the genus *Bacillus*. Three sequences with a similarity level of about 95% belonged to non-cultured *Firmicutes* from the thermophilic microbial fuel cell^27^, and the other 16S rRNA gene sequences in the GeneBank had an identity of less than 90%. Thus AL32 represented a novel species and genus, which we designated ‘*Candidatus* Carbobacillus altaicus’. Like *B. lithotrophica*, AL32 is a rarely detected phylogenetically isolated lineage of *Firmicutes* (Fig. 4).

Analysis of the genome of *Ca*. Carbobacillus altaicus AL32 revealed a complete electron transport chain typical for aerobes and including proton-translocating NADH: quinoine oxidoreductase-like complex, membrane-bound succinate dehydrogenase, quinone-cytochrome C reductase (complex III) and terminal oxygen reductases (Fig. 5). The transmembrane ion gradient generated by an F_0_F_1_- type ATPase may be used for ATP synthesis. The first complex contains the subunits similar to NuoA, B, C, D, H, I, J, K, L, M, and N while the subunits NuoEFG that forms NADH-interacting module were not found, indicating that NADH is likely not an actual electron donor. Terminal oxygen reductases in *Ca*. Carbobacillus altaicus AL32 are represented by a cc*(o/b)a*_3_ - type oxidase, *ba*_3_ – type of heme-copper cytochrome/quinol oxidase, and a quinol oxidase *bd* complex. The first operon is followed by a heme A synthase and protoheme IX farnesyltransferase necessary to make haem *o* from haem *b* presurcor. All cytochrome oxidase genes have close homologues in the genomes of aerobic bacilli, including the closest relative of *Ca*. Carbobacillus altaicus AL32, *H. schlegelii*^28^. The cytochrome oxidases of these types differ in their affinity for oxygen^29^ thus their simultaneous presence may allow respiration of *Ca*. Carbobacillus altaicus AL32 under a wider range of microaerophilic and aerobic conditions.

In the genome of *Ca*. Carbobacillus altaicus AL32 there is a set of genes for Embden-Meyerhof pathway and special enzymes that carry out gluconeogenesis reactions, with the exception of phosphoglycerate mutase that could be missed in the assembly. Pentose phosphate pathway is also present, with the exception of the phosphogluconolactonase gene, which was not detected also in *B. lithotrophica* AL31. Unlike *B. lithotrophica* AL31, decarboxylation of pyruvate to acetyl-CoA in *Ca*. Carbobacillus altaicus AL32 could be carried out by а pyruvate dehydrogenase. Subsequent conversion of acetyl-CoA to acetate with ATP synthesis can be carried out by acetyl-CoA synthetase. Next to acetyl-CoA synthetase are the genes of the acetate permease transporter. The tricarboxylic acid (TCA) cycle in *Ca*. Carbobacillus altaicus AL32 is complete enabling oxidation of organic substrates in course of aerobic or anaerobic respiration. The known pathways for autotrophic carbon fixation, in particular, the Wood-Ljungdahl pathway and the Calvin cycle are absent, and the possibility of using the reverse tricarboxylic acid cycle is also unlikely, since we did not find the key enzyme of this pathway, citrate lyase. Possible substrates for heterotrophic growth of *Ca*. Carbobacillus altaicus AL32, in addition to low-molecular weight sugars and peptides, can be fatty acids, as evidenced by the presence of beta-oxidation pathway, including esterase, fatty-acid-CoA ligase, acyl-CoA dehydrogenase, 3-hydroxyacyl-CoA dehydrogenase/ enoyl-CoA hydratase, and 3-ketoacyl-CoA thiolase. Propionyl-CoA, formed as a product of beta-oxidation of odd-chain fatty acids, could be metabolised in methylcitrate cycle, including methylcitrate synthase, methylcitrate dehydratase, 2-methylisocitrate lyase and TCA enzymes.

The ability of *Ca*. Carbobacillus altaicus AL32 to use molecular hydrogen as an energy source is indicated by the presence of membrane-bound [NiFe] -family group 1d hydrogenase. This group comprises oxygen-tolerant uptake hydrogenases providing electron input for aerobic respiration and oxygen-tolerant anaerobic respiration^30^. In addition to this enzyme, there is a multi-subunit membrane-bound hydrogenase of group 4 f. Such multimeric enzymes could couple oxidation of a C1 compounds like formate to proton reduction concurrent with proton translocation^30^. The presence of this hydrogenase is consistent with the presence of the formate dehydrogenase. The presence of the N-terminal Tat signal peptide in the catalytic subunit suggests that this complex is localized on the outer side of the cytoplasmic membrane.

The search for potential terminal oxidoreductases of anaerobic respiratory pathways revealed the presence of two membrane-bound molybdopterin oxidoreductases of the Psr/Psh family. Phylogenetic analysis of the catalytic subunit of one of them showed that it is likely a tetrathionate reductase (Supplemental Fig. S2). Specificity of the second enzyme could not be reliably predicted based on its amino acid sequence. The pathways of dissimilatory reduction of nitrate, nitrite and sulfate were not detected.

We propose the following taxonomic names for the novel genus and species of AL32:

‘*Candidatus* Carbobacillus’ gen. nov.

‘Candidatus Carbobacillus altaicus gen. et sp. nov.

*Etymology*. Carbobacillus (car.bo.ba.cil’lus L. masc. n. carbo coal; L. masc. n. bacillus a rod; L. masc. n. Carbobacillus a rod from a coal deposit). Carbobacillus altaicus (al.ta’i.cus N.L. masc. adj. altaicus, originating from Altay).

### *H. schlegelii* AL33: aerobic hydrogenotrophic facultative chemolithoautotroph

The genome of the third most numerous member of the microbial community, *H. schlegelii* AL33, was obtained as 110 contigs with a total length of 2,735,157 nt. CheckM estimates the completeness of this genome at 94% with 0.8% contamination and 20% strain heterogeneity. The 16S rRNA sequence corresponding to this bin had a 99.5% identity with the 16S rRNA of the *H. schlegelii* strain MA48, a 2.61 Mbp-long draft genome of which was recently sequenced^28^. The value of DNA-DNA hybridization *in silico* of two genomes was about 92%, which confirms assignment of the strain AL33 to the species *H. schlegelii*. Differences between two genomes are mainly related to genes encoding hypothetical proteins and putative phage-related regions.

According to the original description, *Bacillus schlegelii* MA48 (later reclassified as *H. schlegelii*)^17^ is a spore-forming facultative chemolithoautotroph that could couple hydrogen oxidation to aerobic respiration^16^. It can also grow chemo-organotrophically on some amino acids, organic acids and aromatic compounds^16^. The ability of *H. schlegelii* strains MA48 and AL33 to use aromatic compounds is indicated by the presence in their genomes of the corresponding catabolic pathways, including a phenylacetate metabolic gene cluster^31^. *H. schlegelii* AL33, as well as *B. lithotrophica* AL31 and *Ca*. Carbobacillus altaicus AL32, are likely spore-forming bacteria, as suggested by the presence of sporulation genes in their genomes (Supplemental Table S3). The genome analysis of *H. schlegelii* AL33 revealed a set of genes required for aerobic respiration: two NADH dehydrogenase-like complexes lacking NuoEFG subunits, succinate dehydrogenase (complex II), cytochrome *bc* complex III, and terminal oxygen reductases. Among the latter there were two *ba*_3_ - type cytochrome *c* oxidase, an cc*(o/b)a*_3_ - type cytochrome *c* oxidase, and a quinol *bd* oxidase. The same components of the aerobic electron transport chain were also found in the genomes of *H. schlegelii* strain MA48^28^, and *Ca*. Carbobacillus altaicus AL32. Complete oxidation of organic substrates can be carried out in TCA, the full set of enzymes of which is encoded in the genome.

The use of hydrogen as an energy source can be enabled by membrane-bound [NiFe] uptake hydrogenase of group 1d^30^. In contrast to the genomes *B. lithotrophica* AL31 and *Ca*. Carbobacillus altaicus AL32, in the genome of *H. schlegelii* AL33 we also detected carbon monoxide dehydrogenase, which can ensure the use of CO, although growth on this substrate has not been tested^16^. The presence of respiratory nitrate reductase is consistent with the ability of the strain *H. schlegelii* MA48 to reduce nitrate to nitrite, although it can not grow anaerobically with nitrate^16^.

Analysis of possible ways of autotrophic fixation of carbon dioxide revealed the presence of the Calvin cycle, while the reverse tricarboxylic acid cycle and the Wood-Ljungdahl pathway were not found. Phylogenetic analysis of the sequence of the large RuBisCO subunit showed that it belongs to the same RuBisCO group (type IE) as the enzymes of other thermophilic autotrophic *Firmicutes*, including *B. lithotrophica* AL31 (Fig. 6).

## Discussion

In this work, we investigated the microbial communities of near-surface coal layer in an open quarry for the mining of brown coal, heated by underground coal combustion. Analysis of the composition of the microbial community of the coal (AL3T) both by 16S rRNA profiling and by metagenomic analysis revealed the presence of only a few dominant groups of microorganisms, almost all of which belonging to the phylum *Firmicutes*. The detected bacterial lineages differed in relation to oxygen requirements and the use of carbon sources. The most abundant group, *Ca*. Carbobacillus altaicus AL32, as could be proposed on the basis of genome analysis, is an aerobic heterotroph growing on low-molecular organic compounds. Pathways of autotrophic carbon fixation in its genome were not detected. The second most abundant microorganism, *B. lithotrophica* AL31, was predicted to thrive anaerobically performing autotrophic CO_2_ assimilation in the Calvin cycle. *H. schlegelii* AL33 is likely an aerobic facultitatively chemolithoautotrophic organism capable of both growth on organic compounds and carrying out CO_2_ fixation of in the Calvin cycle. The same groups of *Firmicutes* were found in a bitumen sample (AL3B), however, the composition of the community was shifted towards autotrophic microorganisms. Thus, in the bitumen sample, *B. lithotrophica* AL31 accounted for the absolute majority (more than 60%) of microorganisms, and the second most abundant group was *H. schlegelii* AL33, while *Ca*. Carbobacillus altaicus AL32 represented less than 1.5%. Probably, such differences can be mainly due to the anaerobic conditions of semi-liquid bituminous substance and less availability of organic substrates that could support the growth of heterotrophs.

A common feature of the metabolism of all three *Firmicutes* species dominating in the communities is their predicted ability to use molecular hydrogen as an energy source, oxidizing it in the processes of aerobic (*Ca*. Carbobacillus altaicus AL32 and *H. schlegelii* AL33) or anaerobic (*B. lithotrophica* AL31) respiration, in the latter case - using sulfur compounds as an electron acceptor. Elemental sulfur and various sulfur-containing minerals detected in the AL3T sample and in the fumarole crust AL3crust were probably carried to the surface with a gas stream. Probably, the processes of underground coal combustion in combination with water supply from the surface create the necessary conditions for processes similar to gasification of coal in course of the production of syngas: 3 C (coal) + O_2_ + H_2_O → H_2_ + 3CO^32^. Molecular hydrogen can be further formed from coal gas in water gas shift reaction: CO + H_2_O → CO_2_ + H_2_. Thus, the presence of hydrogen and CO_2_ in the gases that escape to the surface creates optimal conditions for the development of hydrogenotrophic chemolithoautotrophs that use them as energy and carbon sources. Another high-energy component of the gas, carbon monoxide, can be used by *H. schlegelii* AL33, which genome encodes carbon monoxide dehydrogenase. It is interesting to note the presence in the microbial communities of bacteria close to *Bacillus methanolicus*, an aerobic thermophilic methanol oxidizer. Besides methanol, *B. methanolicus* can utilize only a few carbon sources for energy and growth^21^. Methanol can be formed from syngas components in the reaction CO + 2H_2_ → CH_3_OH.

Two species, *B. lithotrophica* AL31 and *H. schlegelii* AL33 contains all enzymes of the Calvin cycle enabling the autotrophic growth. The key enzyme of this cycle, RuBisCO, belongs to rarely occurring type 1-E. This type of RuBisCO was recently found in metagenomes of microbial communities in Antarctic surface soil, and assigned to the members of *Actinobacteria*, WPS-2, and AD3^33^. It was proposed that these microorganisms grow chemolithoautotrophically, oxidising CO and H_2_ present in the atmosphere at trace concentrations^33^. It is possible that type 1-E RuBisCO is adapted to CO_2_ fixation in hydrogenotrophic bacteria and horizontally transmitted in such ecosystems.

Thermal ecosystems associated with underground coal combustion sites are rare and less studied than geothermal features such as terrestrial hot springs, deep sea hydrothermal vents and other sites associated with volcanic activity. Microbial communities of heated soils around coal-fire gas vents in Xinjiang, China, were studied by T-RFLP analysis and clone libraries of 16S rRNA genes^4^. These microbial communities comprised the members of the phyla *Firmicutes, Proteobacteria, Acidobacteria, Bacteroidetes, Planctomycetes* and *Actinobacteria*; Archaea were also found. *Firmicutes* were the most numerous group, but the detected phylotypes were phylogenetically distant from those found in our work. Perhaps differences in the composition of microbial communities may be due to the fact that Zhang *et al*.^4^ examined soils samples rich in organic carbon, while in our work the object of investigation was the coal located on the surface, on which a specialized community of microorganisms, driven by coal gases, developed.

It is interesting that from the whole variety of thermophilic microorganisms capable of oxidizing hydrogen, including representatives of the phyla *Aquificae, Proteobacteria, Firmicutes, Thermodesulfobacteria*, we detected only few lineages of *Firmicutes*. Phylogenetically closely related bacteria were found both in geothermal environments (*B. lithotrophica, ‘B. solfatarensis’, K. tusciae*), and in mesophilic ecosystems. Thus, various strains of *H. schlegelii* were isolated both from lake sediments in Switzerland^16^ and from volcanic soils in Antarctica^34^. It was suggested that the strains of *H. schlegelii* found in cold environments are allochthonous, and their origin is in geothermal sites^35^. The object we studied is a young thermal ecosystem, formed only several years or few decades ago. We propose that thermophilic *Firmicutes*, whose spores can spread from their original geothermal habitats over long distances, are the first colonizers of this new thermal ecological niche.

## Methods

### Sampling, field measurements and chemical analyses

The samples were collected on 9 August 2015 from three different spots in the open pit. A composite sample designated AL3T was collected from five different locations in the close vicinity of the fumarole-like structure. The upper layer (1–10 cm) of the soil (AL3T) or bituminous substance (AL3B) was sampled into sterile 50 ml plastic tubes. Contents of the tubes of AL3T were aseptically pooled in order to minimize spatial variations. The white color crust on the fumarole surface was collected for the elemental analysis and designated AL3crust. All samples were kept at +4 °C before analysis.

The mineralogical composition and elemental composition of the AL3T, AL3B, and AL3crust samples were characterized by X-ray diffraction (XRD) using a Shimadzu XRD-6000 (Shimadzu Corporation, Kyoto Japan) diffractometer and scanning electron microscopy (SEM) using Philips SEM 515 (Philips Electronic Instruments, Eindhoven, The Netherlands) with energy dispersive spectrometry (EDS; EDAX Inc., Mahwan, NJ) as previously described^36^.

### 16S rRNA gene sequencing and analysis

DNA from AL3T and AL3B samples was isolated using the MO BIO Power Soil DNA Kit (MO BIO Laboratories, Qiagen Inc., Valencia, CA). The “universal” primers U341F (5′-CCT ACG GGR SGC AGC AG) and U806R (5′-GGA CTA CYV GGG TAT CTA AT) were used for amplification of 16S RNA gene fragments. PCR amplification was performed using GoTaq polymerase (Promega, USA). PCR conditions were 96 °C for 2 min, 30 cycles of 96 °C for 40 s, 58 °C for 40 s, 72 °C for 60 s, and a final step of elongation at 72 °C for 10 min. The PCR fragments were sequenced with a Roche Genome Sequencer (GS FLX), using the Titanium XL + protocol according to the manufacturer’s instructions. A total of 11025 and 4823 reads were generated for samples AL3T and AL3B, respectively. Short reads, reads with mismatches to primer sequences as well as those containing ambiguous nucleotides were excluded from analysis. Chimeric sequences were detected and removed by UCHIME^37^. Singleton sequences were removed prior to OTU clustering. Clustering and selection of representative sequences for operational taxonomic units (OTUs) was done using RDP mcClust^38^. OTUs were assigned to taxonomic groups based on BLASTN searches against the NCBI database. OTUs were assigned to a certain species if the representative sequence was more than 97% identical to that of the 16S rRNA gene of a cultivated microorganism. If the sequence had an identity of more than 97% with the 16S rRNA genes of several species, then it was classified only at the genus level. Taxonomic assignment of OTUs was also performed using the RDP Classifier^38^.

### Metagenome sequencing and assembly, contig binning, and analysis of the composite genomes

Metagenomic DNA of sample AL3T was fragmented by sonication and the sequencing library was constructed with NEBNext® Ultra™ II DNA Library Prep Kit for Illumina (New England Biolabs, USA) according to the manufacturer’s instructions. The library was sequenced in a single read mode (250 bp) using the Illumina HiSeq. 2500 platform according to the manufacturer’s instructions (Illumina Inc., USA). Sequencing resulted in a total of 15 million high quality sequencing reads after primer and quality trimming (q 30) with Cutadapt^39^ and Sickle (https://github.com/najoshi/sickle), respectively. The assembly of the contigs was carried out using SPAdes Genome Assembler^40^ (specifying -meta parameter indicating metagenome assembly).

Binning of the obtained metagenomic contigs into clusters representing the composite genomes of microbial community members was performed using the program CONCOCT^41^. Completeness, contamination, strain heterogeneity, and preliminary phylogenetic assignment of obtained composite genomes were assessed with CheckM^22^. For each composite genome, gene search and annotation were performed for all contigs longer than 2000 bp, using the RAST server^42^, followed by manual correction by searching the National Center for Biotechnology Information (NCBI) databases. The 16S ribosomal RNA genes were found in contigs by CheckM. The search for continuations of contigs representing fragments of 16S rRNA genes using Bandage program^43^ allowed to link 16S rRNA genes to genome bins where 16S rRNA genes were missing.

Signal peptides were predicted using Signal P v.4.1 for Gram-positive bacteria (http://www.cbs.dtu.dk/services/SignalP/). The N-terminal twin-arginine translocation (Tat) signal peptides were predicted using PRED-TAT (http://www.compgen.org/tools/PRED-TAT/) and the transmembrane helices with TMHMM Server v. 2.0 (http://www.cbs.dtu.dk/services/TMHMM/).

The values of DNA-DNA hybridization *in silico* were calculated using GGDC 2^44^, available at http://ggdc.dsmz.de/.

### Phylogenetic analysis

CheckM was used to find 43 single copy marker genes in our assembled genomes and genomes of several other *Firmicutes*. Multiple alignment of concatenated amino acid sequences of marker genes from these genomes and from all species presented in CheckM database was constructed using CheckM. Selected part of the CheckM multiple alignment, comprising 3 newly assembled genomes, 58 members of *Firmucutes*, and *Aquifex aeolicus* was used for the construction of maximum likelihood phylogenetic tree in PhyML^45^ using default parameters. The support values for the internal nodes were estimated by approximate Bayesian tests in PhyML. *Aquifex aeolicus* was used to root the tree.

For phylogenetic analysis of RbcL (the large subunit of RuBisCO) proteins the amino acid sequences were aligned using MUSCLE included in MEGA 6.0^46^. The evolutionary history was inferred by using the Maximum Likelihood method based on the JTT matrix-based model. All positions containing gaps and missing data were eliminated. There were a total of 424 positions in the final dataset. Evolutionary analyses were conducted in MEGA 6.0. The support values for the internal nodes were estimated from 100 bootstrap replicates. RbcL from *Rhodospirillum rubrum* P04718 was used to root the tree.

### Nucleotide sequence accession number

Pyrosequencing read data obtained for the 16S rRNA gene fragments were deposited in Sequence Read Archive (SRA) under the accession numbers SRR5966983 (AL3T), and SRR5966982 (AL3B).

The annotated genome sequence of *Brockia lithotrophica* AL31, *Ca*. Carbobacillus altaicus AL32, and *Hydrogenibacillus schlegelii* AL33 has been deposited in the GenBank database under accession numbers PEBW00000000, PEBX00000000, and PEBV00000000, respectively. The gene numbers mentioned in the supplemental material correspond to these annotations.

## Electronic supplementary material

Supplementary Information

## References

[1] Stracher GB, Taylor TP (2004). Coal fires burning out of control around the world: thermodynamic recipe for environmental catastrophe. Int. J. Coal Geol..

[2] Rattigan JH (1967). Phenomena about Burning Mountain, Wingen, N.S.W. Aust. J. Earth Sciences.

[3] Engle MA (2012). Gas emissions, minerals, and tars associated with three coal fires, Powder River Basin, *USA*. Sci. Total Environ..

[4] Zhang T, Xu J, Zeng J, Lou K (2013). Diversity of prokaryotes associated with soils around coal-fire gas vents in MaNasi county of Xinjiang, China. Antonie Van Leeuwenhoek..

[5] Pone JDN (2007). The spontaneous combustion of coal and its by products in the Witbank and Sasolburg coalfields of South Africa. Int. J. Coal Geol..

[6] Tammy TJ (2005). Nitrogen changes and domain bacteria ribotype diversity in soils overlying the Centralia, Pennsylvania underground coal mine fire. Soil Sci..

[7] Gribenski N (2016). Complex patterns of glacier advances during the late glacial in the Chagan Uzun Valley, Russian Altai. Quaternary Science Reviews..

[8] Baker VR, Benito G, Rudoy AN (1993). Paleohydrology of late pleistocene superflooding, Altay Mountains, Siberia. Science.

[9] Agatova AR, Nepope RK (2011). Assessing the rate of seismogravitational denudation of the relief of southeastern Altai: The Chagan-Uzun R. Basin. J. Volcanol. Seismolog..

[10] Nepop, R. K. & Agatova, A. R. Earthquake induced landslides in Russian Altai: absolute dating applying tree-ring and radiocarbon analysis. In *Advancing Culture ofLiving with Landslides*. (eds Mikoš, M., Casagli, N., Yin, Y., Sassa, K.) (Springer, Cham, 2017) (2017).

[11] Deviatkin, E. V. Cenozoic deposits and neotectonics in south-eastern Altai. *Proceedings of the Geological Institute of the USSR Academy of Science*. V. 126, 285 pp. (Nauka Publishers, Moscow, 1965).

[12] Ehrlich H.L. & Newman D.K. *Geomicrobiology* 5^th^ ed. (Taylor and Francis Ltd., 2009).

[13] Ludwig, W., Schleifer, K.-H. & Whitman, W. B. Revised road map to the phylum *Firmicutes*. In *Bergey’s Manual of Systematic Bacteriology. Vol. 3* (Springer-Verlag, 2008).

[14] Perevalova AA, Kublanov IV, Baslerov RV, Zhang G, Bonch-Osmolovskaya EA (2013). *Brockia lithotrophica* gen. nov., sp. nov., an anaerobic thermophilic bacterium from a terrestrial hot spring. Int. J. Syst. Evol. Microbiol..

[15] Stackebrandt, E. The Family *Thermoanaerobacteraceae*. In *The Prokaryotes* (eds. Rosenberg, E., DeLong, E. F., Lory, S., Stackebrandt, E., Thompson, F.) 413–419 (Springer, 2014).

[16] Schenk A, Aragno M (1979). *Bacillus schlegelii*, a new species of thermophilic, facultatively chemolithoautotrophic bacterium oxidizing molecular hydrogen. J. Gen. Microbiol..

[17] Kämpfer P, Glaeser SP, Busse HJ (2013). Transfer of *Bacillus schlegelii* to a novel genus and proposal of *Hydrogenibacillus schlegelii* gen. nov., comb. nov. Int. J. Syst. Evol. Microbiol..

[18] Bonjour F, Aragno M (1984). *Bacillus tusciae*, a new species of thermoacidophilic, facultatively chemoautotrophic, hydrogen oxidizing sporeformer from a geothermal area. Arch. Microbiol..

[19] Klenk HP (2011). Complete genome sequence of the thermophilic, hydrogen-oxidizing *Bacillus tusciae* type strain (T2) and reclassification in the new genus, *Kyrpidia* gen. nov. as *Kyrpidia tusciae* comb. nov. and emendation of the family *Alicyclobacillaceae* da Costa and Rainey, 2010. Stand. Genomic Sci..

[20] Arfman N (1992). *Bacillus methanolicus* sp. nov., a new species of thermotolerant, methanol-utilizing, endospore-forming bacteria. Int. J. Syst. Bacteriol..

[21] Brautaset T, Jakobsen M, Josefsen KD, Flickinger MC, Ellingsen TE (2007). *Bacillus methanolicus*: a candidate for industrial production of amino acids from methanol at 50 degrees C. Appl. Microbiol. Biotechnol..

[22] Parks DH, Imelfort M, Skennerton CT, Hugenholtz P, Tyson GW (2015). CheckM: assessing the quality of microbial genomes recovered from isolates, single cells, and metagenomes. Genome Res..

[23] Bowers RM (2017). Minimum information about a single amplified genome (MISAG) and a metagenome-assembled genome (MIMAG) of bacteria and archaea. Nat. Biotechnol..

[24] Park SW (2009). Presence of duplicate genes encoding a phylogenetically new subgroup of form I ribulose 1,5-bisphosphate carboxylase/oxygenase in *Mycobacterium* sp. strain JC1 DSM 3803. Res Microbiol..

[25] Vignais PM, Billoud B (2007). Occurrence, classification, and biological function of hydrogenases: an overview. Chem. Rev..

[26] Rothery RA, Workun GJ, Weiner JH (2008). The prokaryotic complex iron-sulfur molybdoenzyme family. Biochim. Biophys. Acta.

[27] Wrighton KC (2008). A novel ecological role of the *Firmicutes* identified in thermophilic microbial fuel cells. ISME J..

[28] Maker A, Hemp J, Pace LA, Ward LM, Fischer WW (2017). Draft genome sequence of *Hydrogenibacillus schlegelii* MA48, a deep-branching member of the *Bacilli* class of *Firmicutes*. Genome Announc..

[29] Ekici S, Pawlik G, Lohmeyer E, Koch HG, Daldal F (2012). Biogenesis of cbb(3)-type cytochrome c oxidase in *Rhodobacter capsulatus*. Biochim. Biophys. Acta.

[30] Greening C (2016). Genomic and metagenomic surveys of hydrogenase distribution indicate H2 is a widely utilised energy source for microbial growth and survival. ISME J..

[31] Teufel R (2010). Bacterial phenylalanine and phenylacetate catabolic pathway revealed. Proc. Natl. Acad. Sci. USA.

[32] Shafirovich E, Varma A (2009). Underground coal gasification: a brief review of current status. Ind. Eng. Chem. Res..

[33] Ji M (2017). Atmospheric trace gases support primary production in Antarctic desert surface soil. Nature.

[34] Andrew Hudson JA, Daniel RM, Morgan HW (1988). Isolation of a strain of *Bacillus schlegelii* from geothermally heated Antarctic soil. FEMS Microbiol. Lett..

[35] Bonjour F, Graber A, Aragno M (1988). Isolation of *Bacillus schlegelii*, a thermophilic, hydrogen oxidizing, aerobic autotroph, from geothermal and nongeothermal environments. Microb. Ecol..

[36] Ikkert OP, Gerasimchuk AL, Bukhtiyarova PA, Tuovinen OH, Karnachuk OV (2013). Characterization of precipitates formed by H2S-producing, Cu-resistant Firmicute isolates of *Tissierella* from human gut and *Desulfosporosinus* from mine waste. Antonie van Leeuwenhoek.

[37] Edgar RC, Haas BJ, Clemente JC, Quince C, Knight R (2011). UCHIME improves sensitivity and speed of chimera detection. Bioinformatics.

[38] Cole JR (2009). The Ribosomal Database Project: improved alignments and new tools for rRNA analysis. Nucleic Acids Res..

[39] Martin M (2011). Cutadapt removes adapter sequences from high-throughput sequencing reads. EMBnet journal..

[40] Bankevich A (2012). SPAdes: a new genome assembly algorithm and its applications to single-cell sequencing. J. Comput. Biol..

[41] Alneberg J (2014). Binning metagenomic contigs by coverage and composition. Nat. Methods..

[42] Brettin T (2015). RASTtk: a modular and extensible implementation of the RAST algorithm for building custom annotation pipelines and annotating batches of genomes. Sci. Rep..

[43] Wick RR, Schultz MB, Zobel J, Holt KE (2015). Bandage: interactive visualisation of de novo genome assemblies. Bioinformatics.

[44] Meier-Kolthoff JP, Auch AF, Klenk H-P, Göker M (2013). Genome sequence-based species delimitation with confidence intervals and improved distance functions. BMC Bioinformatics.

[45] Guindon S (2010). New algorithms and methods to estimate maximum-likelihood phylogenies: assessing the performance of PhyML 3.0. Syst. Biol..

[46] Tamura K, Stecher G, Peterson D, Filipski A, Kumar S (2013). MEGA6: Molecular Evolutionary Genetics Analysis version 6.0. Mol. Biol. Evol..

