# Supplementary material for: Lignite coal burning seam in the remote Altai Mountains harbors a hydrogen-driven thermophilic microbial community
Source: Sci Rep. 2018 Apr 30;8:6730. doi: 10.1038/s41598-018-25146-9 (PMC5928048; doi:10.1038/s41598-018-25146-9)
Supplement: Supplementary file 1 — Supplementary Information [file 41598_2018_25146_MOESM1_ESM.pdf]

Supplemental material

Lignite coal burning seam in the remote Altai Mountains harbors a hydrogen-driven thermophilic microbial community

Vitaly V. Kadnikov, Andrey V. Mardanov, Denis A. Ivasenko, Dmitry V. Antsiferov, Alexey V. Beletsky, Olga V. Karnachuk, and Nikolay V. Ravin

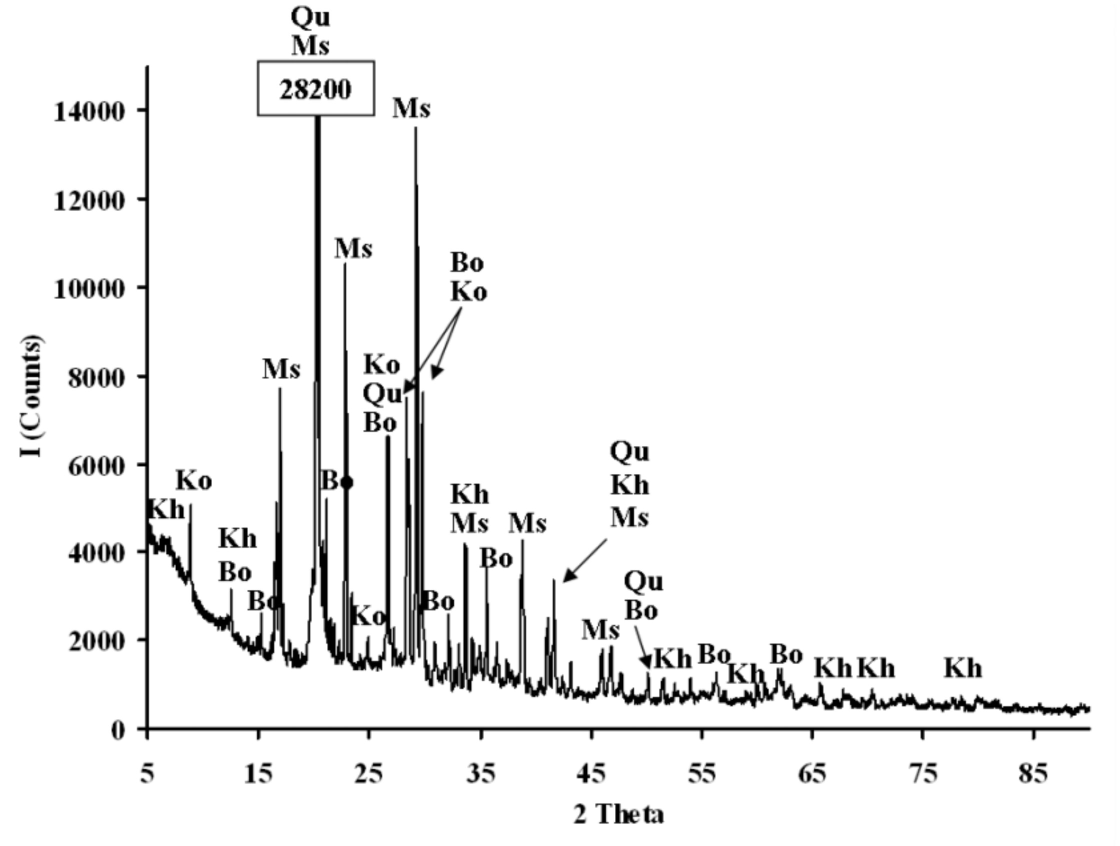

Figure S1. X-ray diffraction analysis of sample Al3crust

Designation of minerals: Bo, boussingaultite $(NH_4)_2(Mg(H_2O)_6)(SO_4)_2)$; Ms, mascagnite $(NH_4)_2SO_4)$; Ko, koktaite $(NH_4)_2Ca(SO_4)_{2*}(H_2O)$; Qu, quartz (SiO_2); Kh, clinochlore (Mg, Fe, $Al)_6(Si,Al)_4O_{10}(OH)_8)$.

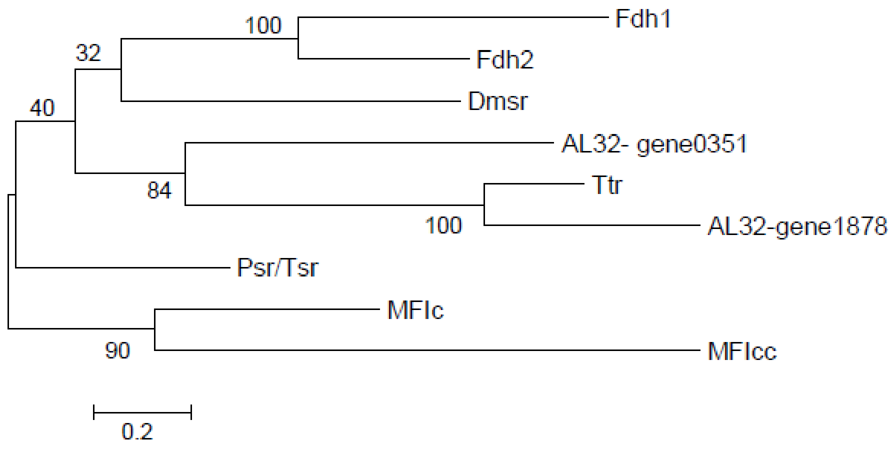

Figure S2. Phylogenetic tree of the catalytic A subunits of molybdopterin oxidoreductases of *Ca.* Carbobacillus altaicus AL32

For phylogenetic analysis of the catalytic A subunits of molybdopterin oxidoreductases predicted protein products of genes 351 and 1878 were used along with consensus sequences of the A subunits of tetrathionate reductases (Ttr), formate dehydrogenases (Fdh1 and Fdh2), thiosulfate or polysulfide reductases (Psr), and DMSO reductases (Dmsr), MFIcc complex (MFIcc), and MFIc complex (MFIc) defined in (Yanyushin et al., Biochemistry. 2005; 44(30): 10037-45). Amino acid sequences were aligned using MUSCLE, ambiguously aligned sites were removed using trimAl before the phylogenetic reconstruction. The maximum likelihood phylogenetic tree was computed by PhyML 3.1, using the gamma model of rate heterogeneity (four discrete rate categories, an estimated alpha-parameter) and LG substitution matrix. Numbers at nodes represent the support values estimated by an approximate Bayesian method. The scale bar represents the average number of substitutions per site.

Table S1. Relative abundance of taxonomic groups

Taxonomic assignment	AL3T	AL3B
Ca. Carbobacillus altaicus AL32 *	46,6%	1,4%
Brockia lithotrophica AL31	14,7%	63,5%
Hydrogenibacillus schlegelii AL33	3,7%	22,0%
Thermoanaerobacter sp.	5,8%	5,0%
Bacillus methanolicus	2,8%	1,5%
Kyrpidia tusciae	0,8%	<0.1%
Desulfotomaculum sp.	0,2%	<0.1%
Other *Firmicutes* (uncultured groups)	23,5%	6,5%
Proteobacteria	1,9%	0,1%
**Total**	**100,0%**	**100,0%**

* lineage close to unvalidated species '*Bacillus solfatarensis*'

Table S2. Genes of *Brockia lithotrophica* AL31, *Ca.* Carbobacillus altaicus AL32, and *Hydrogenibacillus schlegelii* AL33 discussed in the text.

Gene No	Predicted protein
	***Brockia lithotrophica* AL31**
442	glucokinase
1439	glucose-6-phosphate isomerase
253	6-phosphofructokinase
435	fructose bisphosphate aldolase
1447	triose phosphate isomerase
241, 1449	glyceraldehyde-3 phosphate dehydrogenase
1448	3-phosphoglycerate kinase
1446	phosphoglycerate mutase
1445	enolase
252	pyruvate kinase
920	phosphate dikinase
434	fructose-1,6-bisphosphatase
1415	glucose-6-phosphate 3- dehydrogenase
1231	6-phosphogluconate dehydrogenase
1379	ribulose phosphate 3-epimerase
424	ribose 5-phosphate isomerase
484, 494, 1206	transketolase
495	transaldolase
368-369	pyruvate:ferredoxin oxidoreductase
487	ribulose 1,5-bisphosphate carboxylase, large subunit
586	ribulose 1,5-bisphosphate carboxylase, small subunit
485	phosphoribulokinase
604, 1304	[NiFe] - hydrogenase of group 1, large subunit
603, 1303	[NiFe] - hydrogenase of group 1, small subunit
602, 1302	[NiFe] - hydrogenase of group 1, cytochrome *b* subunits
537	Formate dehydrogenase accessory protein FdhD
538	Formate dehydrogenase, alpha subunit
540	Formate dehydrogenase, beta subunit
541	Formate dehydrogenase, gamma subunit
542	Frmate dehydrogenase accessory protein FdhE
1512	Psr/Psh family oxidoreductase, catalytic A subunit
1511	Psr/Psh family oxidoreductase, iron-sulfur electron transfer B subunit
1509	Psr/Psh family oxidoreductase, membrane C subunit
409	F_0F_1-type ATPase, epsilon chain
410	F_0F_1-type ATPase, beta chain
411	F_0F_1-type ATPase, gamma chain
412	F_0F_1-type ATPase, alpha chain
413	F_0F_1-type ATPase, delta chain
414	F_0F_1-type ATPase, F0 sector subunit b
415	F_0F_1-type ATPase, F0 sector subunit c
416	F_0F_1-type ATPase, F0 sector subunit a

	***Ca.* Carbobacillus altaicus AL32**
2602	NADH-ubiquinone oxidoreductase chain M (EC 1.6.5.3)
2601	NADH-ubiquinone oxidoreductase chain N (EC 1.6.5.3)
2600	NADH-ubiquinone oxidoreductase chain L (EC 1.6.5.3)
1546	NADH-ubiquinone oxidoreductase chain K (EC 1.6.5.3)
1547	NADH-ubiquinone oxidoreductase chain J (EC 1.6.5.3)
1548	NADH-ubiquinone oxidoreductase chain I (EC 1.6.5.3)
1549	NADH-ubiquinone oxidoreductase chain H (EC 1.6.5.3)
1550	NADH-ubiquinone oxidoreductase chain D (EC 1.6.5.3)
1551	NADH-ubiquinone oxidoreductase chain C (EC 1.6.5.3)
1552	NADH-ubiquinone oxidoreductase chain B (EC 1.6.5.3)
1553	NADH ubiquinone oxidoreductase chain A (EC 1.6.5.3)
1951	Citrate synthase
2678	aconitate hydratase
1952	Isocitrate dehydrogenase
215, 1025	2-oxoglutarate oxidoreductase, alpha subunit
214, 1024	2-oxoglutarate oxidoreductase, beta subunit
934	Succinyl-CoA ligase [ADP-forming] alpha chain
935	Succinyl-CoA ligase [ADP-forming] beta chain
398	Succinate dehydrogenase iron-sulfur protein
399	Succinate dehydrogenase flavoprotein subunit
400	Succinate dehydrogenase cytochrome b558 subunit
790	Fumarate hydratase
1953	Malate dehydrogenase
2897	Menaquinone-cytochrome C reductase iron-sulfur subunit
2898	Menaquinone-cytochrome c reductase, cytochrome B subunit
2899	Menaquinone-cytochrome C oxidoreductase, cytochrome C subunit
2900	Menaquinone-cytochrome C oxidoreductase, cytochrome C subunit
1554	F_0F_1-type ATPase, epsilon chain
1555	F_0F_1-type ATPase, beta chain
1556	F_0F_1-type ATPase, gamma chain
1557	F_0F_1-type ATPase, alpha chain
1558	F_0F_1-type ATPase, delta chain
1559	F_0F_1-type ATPase, F0 sector subunit b
1560	F_0F_1-type ATPase, F0 sector subunit c
1561	F_0F_1-type ATPase, F0 sector subunit a
1396	cc*(o/b)*a_3 - type oxidase, subunit II
1397	cc*(o/b)*a_3 - type oxidase, subunit I
1398	cc*(o/b)*a_3 - type oxidase, subunit III
1399	cc*(o/b)*a_3 - type oxidase, subunit IV
1400	cytochrome c oxidase assembly factor CtaG
1401	heme A synthase
1402	protoheme IX farnesyltransferase
1280	ba_3 – type heme-copper cytochrome/quinol oxidase, subunit II
1281	ba_3 – type heme-copper cytochrome/quinol oxidase,

	subunit I
2047	quinol oxidase *bd* complex subunit I
2056	quinol oxidase *bd* complex subunit II
1748	Pyruvate dehydrogenase E1 component alpha subunit
1749	Pyruvate dehydrogenase E1 component beta subunit
1750	Dihydrolipoamide acetyltransferase component of pyruvate dehydrogenase complex
1751	Dihydrolipoamide dehydrogenase of pyruvate dehydrogenase complex
101, 1244	acetyl-CoA synthetase
102, 1245	acetate permease
1644, 2420, 2088	esterase
300, 614, 2815	fatty-acid-CoA ligase
605, 1203	acyl-CoA dehydrogenase
1205	3-hydroxyacyl-CoA dehydrogenase/ enoyl-CoA hydratase
1204, 1589, 1855, 2813	3-ketoacyl-CoA thiolase
1887	methylcitrate synthase
2843	methylcitrate dehydratase
2842	2-methylisocitrate lyase
764	[NiFe] - hydrogenase of group 1d, cytochrome b subunit
763	[NiFe] - hydrogenase of group 1d, large subunit
762	[NiFe] - hydrogenase of group 1d, small subunit
1888	[NiFe] - hydrogenase of group 4f component
1889	[NiFe] - hydrogenase of group 4f component
1890	[NiFe] - hydrogenase of group 4f component
1891	[NiFe] - hydrogenase of group 4f component
1892	[NiFe] - hydrogenase of group 4f component
2635	[NiFe] - hydrogenase of group 4f component
2636	[NiFe] - hydrogenase of group 4f component
2637	[NiFe] - hydrogenase of group 4f component
2638	[NiFe] - hydrogenase of group 4f component
2639	[NiFe] - hydrogenase of group 4f component
2640	[NiFe] - hydrogenase of group 4f component
1782	Formate dehydrogenase assembly factor FdhD
1783	Formate dehydrogenase, alpha subunit (EC 1.2.1.2)
1784	Formate dehydrogenase, beta subunit (EC 1.2.1.2)
1785	Formate dehydrogenase, NrfD-like membrane subunit
1875	Tetrathionate reductase subunit C
1876	Tetrathionate reductase subunit B
1877	Uncharacterized component of anaerobic dehydrogenases, chaperone TorD
1878	Tetrathionate reductase subunit A
351	Psr/Psh family oxidoreductase, catalytic subunit
352	Psr/Psh family oxidoreductase, fused 4Fe-4S and NdfD-like membrane subunits

Hydrogenibacillus schlegelii **AL33**	
448	Phenylacetic acid degradation protein paaI
449	Phenylacetate-coenzyme A ligase
450	Enoyl-CoA hydratase
451	Enoyl-CoA hydratase
452	3-hydroxybutyryl-CoA dehydrogenase precursor
453	3-hydroxybutyryl-CoA dehydrogenase
454	3-ketoacyl-CoA thiolase (EC 2.3.1.16)
455	Phenylacetic acid degradation protein PaaD, thioesterase
456	hypothetical protein
457	Phenylacetic acid degradation operon negative regulatory protein PaaX
458	Phenylacetate-CoA oxygenase, PaaG subunit
459	Phenylacetic acid degradation protein PaaB
460	Phenylacetate-CoA oxygenase, PaaI subunit
461	Phenylacetate-CoA oxygenase, PaaJ subunit
462	dihydrolipoyl dehydrogenase
463	Enoyl-CoA hydratase
1540, 1818	Cytochrome c oxidase (B(O/a)3-type) chain II
1541, 1817	Cytochrome c oxidase (B(O/a)3-type) chain I
1115	cc$(o/b)a_3$ - type oxidase, subunit II
1116	cc$(o/b)a_3$ - type oxidase, subunit I
1117	cc$(o/b)a_3$ - type oxidase, subunit III
1118	cc$(o/b)a_3$ - type oxidase, subunit IV
1729	quinol oxidase *bd* complex subunit I
1728	quinol oxidase *bd* complex subunit II
914	[NiFe] - hydrogenase of group 1d, cytochrome b subunit
913	[NiFe] - hydrogenase of group 1d, large subunit
912	[NiFe] - hydrogenase of group 1d, small subunit
285	Carbon monoxide dehydrogenase medium chain
286	Carbon monoxide dehydrogenase small chain
287	Carbon monoxide dehydrogenase large chain
288	Carbon monoxide dehydrogenase molybdenum cofactor insertion protein CoxF
289	Carbon monoxide oxidation accessory protein CoxG
290	Carbon monoxide dehydrogenase D protein
291	Carbon monoxide dehydrogenase accessory protein CoxE
292	Carbon monoxide dehydrogenase maturation factor CoxF
1143	Respiratory nitrate reductase alpha chain (EC 1.7.99.4)
1144	Respiratory nitrate reductase beta chain (EC 1.7.99.4)
1145	Respiratory nitrate reductase delta chain (EC 1.7.99.4)
1146	Respiratory nitrate reductase gamma chain (EC 1.7.99.4)
1942	ribulose 1,5-bisphosphate carboxylase, large subunit
1943	ribulose 1,5-bisphosphate carboxylase, small subunit

Table S3. The presence of sporulation genes conserved in bacilli and clostridia in the genomes of *Brockia lithotrophica* AL31, *Ca.* Carbobacillus altaicus AL32, and *Hydrogenibacillus schlegelii* AL33.

Gene *	*Brockia lithotrophica* AL31	*Ca.* Carbobacillus altaicus AL32	*Hydrogenibacillus schlegelii* AL33
Stage 0 (pre-septation)			
spo0A	1307	2170	1495
sigH (spo0H)	93	2414	944
spo0J	469	1515	72
obgE	1023	681	1976
Stage II (post-septation)			
spoIIAA	1252	799	1468
spoIIAB	1253	800	1467
sigF (spoIIAC)	1254	2247	1466
spoIID	396	704	160
spoIIE (spoIIH)	127	13	2113
spoIIGA	633	2249	2362
sigE (spoIIGB)	632	2248	2361
spoIIM	-	-	-
spoIIP	1520	-	325
spoIIR	-	1573	127
Stages III-VI (post-engulfment)			
cwlD	42	2360	2600
dacB	1265	1224, 1082	2221
dapA	782	221	774
dapB	956	2903	423
spmA	1266	1223, 1081	2222
spmB	1267	1222, 1080	2223
spoIIIAA	1332	2195	1518
spoIIIAB	1331	2194	1517
spoIIIAC	1330	2193	1516
spoIIIAD	1329	2192	1515
spoIIIAE	1328	2191	1514
spoIIIAF	1327	2190	1513
spoIIIAG	1326	2189	1512
spoIIIAH	1325	2188	1511
spoIIID	394	1740	162
spoIIIE	741	-	-
spoIIIJ	475	2737	66
jag	-	2736	-
sigG (spoIIIG)	631	2247	2360
spoIVA	691	2867	871
spoIVB	1308	2171	1496
sigK	1547	188	722

spoVAC	1437	1407	1127
spoVAD	1436	1408	1128
spoVAEB	1435	1409	1129
spoVB	991	715	2010
pth (spoVC)	137	28	2128
spoVD	645	2260	2374
spoVG	-	31	2131
spoVK	1276	1988	2239
spoVS	723	217	797
spoVT	134	24	2124
stoA (spoIVH)	-	-	1006
yabP	131	18	2118
yabQ	-	17	2117
ylbJ	1128	2664	-
ylmC	630	2246	2358
yqfC	945	2332	264
yqfD	944	2331	265
ytvI	1275	1987	2236
yyaC	-	2517	74
Spore coat			
spoIVA,	691	2867	871
alr (yncD)	364	1020	1415
Germination			
gpr	1521	2517	324
lgt (gerF)	1196	1064	517

* The list of sporulation genes, conserved in bacilli and clostridia, was taken from table 3 in the paper of Galperin et al., Environ Microbiol. 2012; 14(11):2870-90.
